# Incidence and predictors of excessive warfarin anticoagulation in patients with atrial fibrillation—The EWA study

**DOI:** 10.1371/journal.pone.0175975

**Published:** 2017-04-20

**Authors:** Samuli Jaakkola, Ilpo Nuotio, Tuomas O. Kiviniemi, Raine Virtanen, Melina Issakoff, K. E. Juhani Airaksinen

**Affiliations:** 1 Heart Center, Turku University Hospital and University of Turku, Turku, Finland; 2 Department of Acute Internal Medicine, Turku University Hospital and University of Turku, Turku, Finland; 3 Department of Cardiology, Turku City Hospital, Turku, Finland; 4 University of Turku, Turku, Finland; University of Bologna, ITALY

## Abstract

Vitamin K antagonist warfarin is widely used in clinical practice and excessive anticoagulation is a well-known complication of this therapy. Little is known about permanent and temporary predictors for severe overanticoagulation. The aim of this study was to investigate the occurrence and predicting factors for episodes with very high (≥9) international normalized ratio (INR) values in warfarin treated patients with atrial fibrillation (AF). Excessive Warfarin Anticoagulation (EWA) study screened all patients (n = 13618) in the Turku University Hospital region with an INR ≥2 between years 2003–2015. Patients using warfarin anticoagulation for AF with very high (≥9) INR values (EWA Group) were identified (n = 412 patients) and their characteristics were compared to a control group (n = 405) of AF patients with stable INR during long-term follow-up. Over 20% (n = 92) of the EWA patients had more than one event of very high INR and in 105 (25.5%) patients EWA led to a bleeding event. Of the several temporary and permanent EWA risk factors observed, strongest were excessive alcohol consumption in 9.6% of patients (OR 24.4, 95% CI 9.9–50.4, p<0.0001) and reduced renal function (OR 15.2, 95% CI 5.67–40.7, p<0.0001). Recent antibiotic or antifungal medication, recent hospitalization or outpatient clinic visit and the first 6 months of warfarin use were the most significant temporary risk factors for EWA. Excessive warfarin anticoagulation can be predicted with several permanent and temporary clinical risk factors, many of which are modifiable.

## Introduction

Excessive anticoagulation and hemorrhage are well known complications of vitamin K antagonist (VKA) warfarin therapy. High international normalized ratio (INR) level predisposes to significant bleeds and independently increases morbidity and mortality also through mechanisms other than bleeding [1–6]. Although overanticoagulation related mortality can be partly explained with spontaneous INR elevations in critically ill patients, high INR values due to inappropriate warfarin dose adjustments have been reported to increase the risk even more [7]. These findings emphasize the importance of identifying risk factors for high INR values. Since there are no large-scale studies on very high INR values in VKA patients, we sought to evaluate the incidence, patient characteristics and predictors of excessive anticoagulation in patients with atrial fibrillation (AF) on warfarin treatment in a large well-defined patient population.

## Methods

The Excessive Warfarin Anticoagulation (EWA) study (ClinicalTrials.gov Identifier: NCT02761941) is part of a series of research projects addressing anticoagulation and complications related to AF [8, 9]. In this study, we screened with computer searches all INR results between the years 2003 and 2015 from the laboratory database provided by Turku University Hospital laboratory service (TYKSLAB). A total of 13618 patients living in the Turku University Hospital catchment area with at least one INR value of ≥2 were identified. Of the 961431 INR test results in this patient group, 75.0% were ≥2. Excessive warfarin anticoagulation was defined as INR value ≥9 (which is the upper numerical measurement range of the laboratory) at any time during the study period. Consequently, we could identify 412 patients (age ≥18 years) with a prior diagnosis of AF, warfarin anticoagulation and at least one occasion with a very high INR (≥9) value. In patients with multiple events with INR values ≥9, only the first event was included in the present analysis.

To further evaluate baseline characteristics and temporary clinical factors predisposing to excessive anticoagulation a control group of 405 patients with stable anticoagulation was identified from the same database by selecting patients with a diagnosis of AF on long-term (>730 days) warfarin anticoagulation with no high INR (>4) values in regular controls (individual mean interval of INR tests 32 days, maximum interval 60 days). The date with the highest INR value (between 2.7–4.0) was selected as the index date for the data collection. All patients filling these criteria were included in the Control group.

Comprehensive laboratory data were collected through computer searches from the laboratory database. All individual patient records from Turku University Hospital and Turku City Hospital were reviewed using a standardized electronic case report form to collect information on patient characteristics, medication, preceding events, indication for warfarin treatment, clinical manifestation and patient management during the high INR event.

The study protocol was approved by the Medical Ethics Committee of the Hospital District of Southwest Finland. Informed consent was not required, because of the register-based nature of the study. The study complies with the Declaration of Helsinki.

### Definitions

Psychiatric disorder included a diagnosis of depression, bipolar disorder or schizophrenia. Dementia was defined as a diagnosis of Alzheimer’s disease, vascular dementia, Lewy body dementia or mixed dementia regardless of the disease stage. Alcohol abuse consisted of an alcohol related diagnosis or a hospital/health care center visit due to alcohol use. Independent living was defined as living at home independently without outside help in daily routines. Recent antibiotic therapy included any antibiotic drug during the preceding 14 days of the index event date. Liver disease was defined as cirrhosis, hepatitis, cholangitis, metastatic liver nodules, unspecific liver failure, tumor in the liver or fatty liver disease. Estimated glomerular filtration rate (eGFR) was calculated using CKD-EPI (Chronic Kidney Disease Epidemiology Collaboration) equation to classify renal function according to KDIGO (Kidney Disease Improving Global Outcomes) clinical practice guidelines [10].

### Statistical analysis

Continuous variables were reported as mean ± standard deviation if they were normally distributed. Categorical variables were described with absolute and relative (percentage) frequencies. Comparisons between study subgroups were performed with Mann-Whitney U test. Chi-square test and Fisher’s exact test were used for categorical variables. For all variables with more than 0.5% missing data, the exact number of patients with missing data is marked in the table. A binary stepwise logistic regression analysis (backward Wald) was performed to identify independent predictors of very high INR values. Predictors with p<0.05 in the univariate analysis were included in the multivariate analysis. All tests were two-sided and statistical significance was set at 5%. This manuscript was written following STROBE guidelines for the reporting of observational studies [11]. Statistical analyses were performed with SPSS software (version 23.0, SPSS, Inc., Chicago, Illinois) and SAS software (version 9.4, SAS Institute, Inc., Cary, North Carolina).

## Results

We identified 564 patients (4.1% of patients with INR ≥2) with very high INR (≥9) values comprising 0.08% of all INR measurements and 0.10% of INR measurements ≥ 2.0. A total of 412 patients (EWA Group) living in the Turku University Hospital catchment area had AF and at least one very high (≥9) INR value. There were 92 patients (22.3%) in the EWA group with multiple (2 to 5) episodes with INR ≥9. Over half (52.7%) of the patients in the EWA Group had at least one INR value of 5–9 before the index event and 19.7% of the patients in the preceding year.

**Fig 1:**
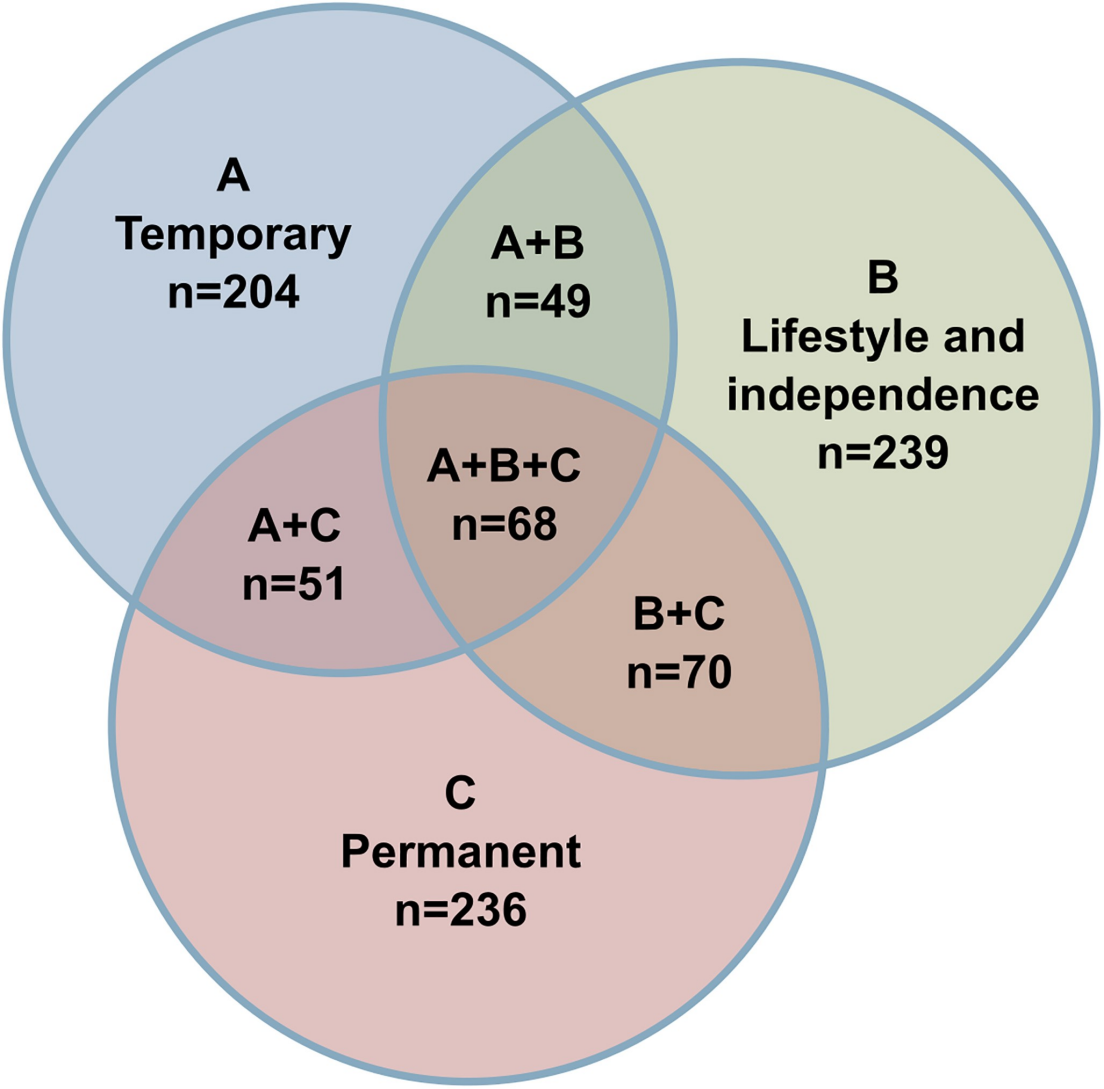
Number of patients with EWA in different risk factor categories. (A) Temporary risk factors = Antibiotic or antifungal therapy, Recent medical treatment (Hospitalization or outpatient visit in the preceding 1 month) or Chemotherapy; (B) Lifestyle and independence related risk factors = Alcohol abuse, Active smoking or non-independence in everyday living; (C) Permanent risk factors = Severe renal dysfunction (eGFR<30), Mechanical heart valve prosthesis, Active malignancy or Chronic heart failure.

Only 105 (25.5%) patients had a significant bleeding related to the very high INR. The baseline characteristics of the patients in the EWA Group and controls with stable warfarin anticoagulation are presented in Table 1. Logistic regression analyses identified several strong independent predictors of very high INR value (Table 2). The number of patients with EWA in different (permanent, temporary or lifestyle related) risk factor categories is illustrated in Fig 1.

**Table 1:** Patient Characteristics.

Patient characteristic	EWA Group (n = 412)	Control Group (n = 405)	P-value
Age (years)	77.7±10.5	76.6±8.5	0.05
Female	217 (52.7)	223 (55.1)	0.493
CHA_2_DS_2_-VASc	4.0 ±1.8	3.7 ± 1.5	0.0007
Chronic heart failure	155 (37.6)	55 (13.6)	<0.0001
Treatment for hypertension	235 (57.0)	283 (70.0)	<0.0001
Diabetes	111 (26.9)	99 (24.4)	0.414
History of ischemic stroke	82 (19.9)	57 (14.1)	0.027
Coronary artery disease	130 (31.6)	89 (22.0)	0.002
History of AMI	80 (19.4)	41 (10.1)	0.0002
Peripheral artery disease	34 (8.3)	9 (2.2)	<0.0001
eGFR^a^			
<15	28 (6.9)	0 (0.0)	<0.0001
15–30	58 (14.2)	5 (1.3)	<0.0001
30–60	148 (36.3)	132 (34.6)	0.614
60–90	114 (27.9)	211 (55.2)	<0.0001
>90	60 (14.7)	34 (8.9)	0.012
Independent living^b^	248 (60.2)	338 (83.7)	<0.0001
Psychiatric disorder	21 (5.1)	11 (2.7)	0.103
Dementia	57 (13.8)	23 (5.7)	<0.0001
History of malignancy	104 (25.2)	53 (13.1)	<0.0001
Active malignancy	48 (11.7)	15 (3.7)	<0.0001
Liver disease	12 (2.9)	1 (0.2)	0.003
Mechanical heart valve	16 (3.9)	1 (0.3)	0.0002
Active smoker	35 (8.5)	7 (1.7)	<0.0001
Alcohol abuse^c^	73 (17.7)	6 (1.5)	<0.0001
Recent bleed^d^	15 (3.6)	4 (1.0)	0.018
Recent surgical operation^e^	30 (7.3)	14 (3.5)	0.016
Recent medical treatment^f^	155 (37.6)	67 (16.5)	<0.0001
Concomitant medication			
Cholesterol-lowering drugs	81 (19.7)	161 (39.8)	<0.0001
NSAID	11 (2.7)	3 (0.7)	0.055
Aspirin	18 (4.4)	9 (2.2)	0.116
SSRI/SNRI	38 (9.2)	11 (2.7)	<0.0001
Tramadol	16 (3.9)	4 (1.0)	0.011
Dronedarone	3 (0.7)	3 (0.7)	1.000
Amiodarone	8 (1.9)	2 (0.5)	0.107
Paracetamole	170 (41.3)	146 (36.0)	0.109
Carbamatzepine	5 (1.2)	2 (0.5)	0.451
Antifungal medication	17 (4.1)	0 (0.0)	<0.0001
Recent antibiotic therapy^g^	107 (26.0)	22 (5.4)	<0.0001
Chemotherapeutic agents	12 (2.9)	3 (0.7)	0.034

Values are mean ± SD, n (%)

AMI = acute myocardial infarction; CHA_2_DS_2_2-VASc = Congestive heart failure, Hypertension, Age 75 (doubled), Diabetes mellitus, prior Stroke, transient ischemic attack or thromboembolism (doubled), Vascular disease, Age 65 to 74, Sex category (female); eGFR = estimated glomerular filtration rate (ml/min/1.73 m2); NSAID = non steroidal anti-inflammatory drug; SSRI/SNRI = selective serotonin/norepinephrine reuptake inhibitor

^a^Data missing on 27 patients (3.3%)

^b^Living at home independently without outside help in daily routines

^c^Alcohol related diagnosis or a hospital/health care center visit due to alcohol use

^d^Bleeding event in the preceding 1 month

^e^Operation in the preceding 1 month

^f^Hospitalization or outpatient visit in the preceding 1 month

^g^Antibiotic therapy in the preceding 14 days

**Table 2:** The Multivariate Predictors of Excessive Warfarin Anticoagulation.

Predictor	Multivariate Analysis OR (95% CI)	P-value
**Temporary**		
Chemotherapeutic agents	5.61 (1.31–24.1)	0.020
Antibiotic or antifungal therapy^a^	4.57 (2.56–8.16)	<0.0001
Recent medical treatment^b^	2.42 (1.56–3.75)	<0.0001
**Lifestyle and independence**		
Alcohol abuse^c^	24.4(9.85–50.4)	<0.0001
Active smoking	3.23 (1.19–8.77)	0.021
Not independent^d^	2.63 (1.73–4.00)	<0.0001
**Permanent**		
Severe renal dysfunction^e^	15.2 (5.70–40.7)	<0.0001
Mechanical heart valve	15.0 (1.74–129)	0.014
Active malignancy	7.07 (3.46–14.4)	<0.0001
Chronic heart failure	2.78 (1.80–4.31)	<0.0001
Treatment for hypertension	0.62 (0.43–0.92)	0.016
Cholesterol-lowering drugs	0.45 (0.30–0.67)	0.001

CI = confidence interval; OR = odds ratio

^a^Antibiotic or antifungal medication in the preceding 14 days.

^b^Hospitalization or outpatient visit in the preceding 1 month

^c^Alcohol related diagnosis or a hospital/health care center visit due to alcohol use

^d^Patient requiring outside help in daily routines

^e^eGFR < 30 ml/min/1.73 m^2^, data missing on 27 patients (3.3%)

### Permanent risk factors

A chronic medical condition predicting the risk of severe overanticoagulation (heart failure, active cancer, severe renal dysfunction and mechanical heart valve prosthesis) was present in 236 patients (57.3%) in the EWA group and in 72 (17.8%) control patients with stable anticoagulation. Only 11.5% of patients (n = 94) had normal eGFR (>90) and almost half (45.4%, n = 371) of the patients had mildly to moderately decreased renal function (eGFR 60–30). Impaired renal function was a significant predictor of EWA, with an odds ratio of over 15 in patients with eGFR<30. EWA was observed in all patients with eGFR < 15.

### Temporary predisposing factors

**Fig 2:**
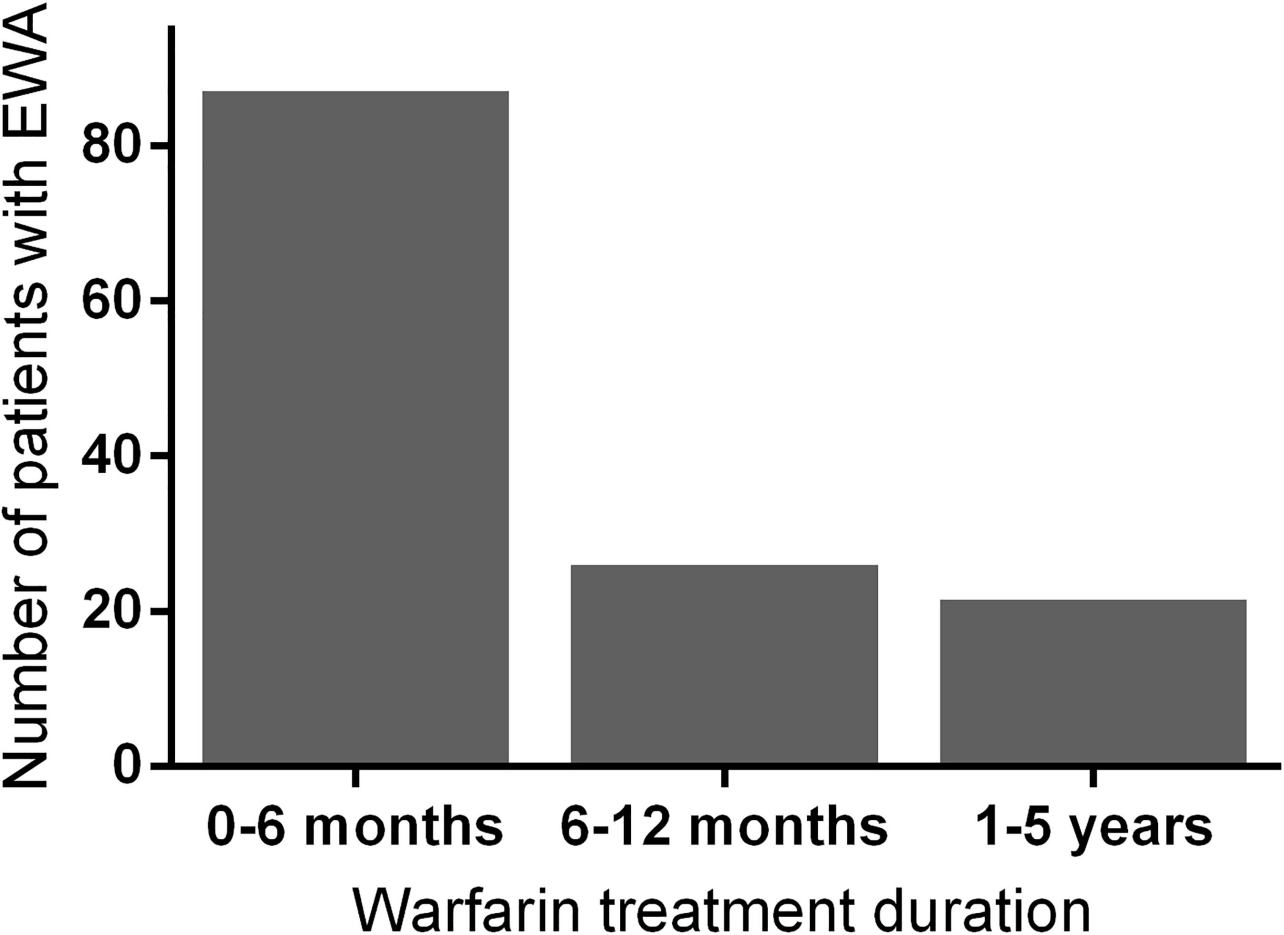
Number of patient with EWA per 6-month episodes from the initiation of warfarin. Data on patients with warfarin treatment duration of more than 5 years are not shown.

A temporary risk factor was found in 204 patients (49.5%) with EWA and 76 control patients (18.8%). Antibiotic or antifungal treatment preceded EWA in 28.6% of patients and caused a 4.6-fold risk for severe overanticoagulation compared to patients with stable INR. An outpatient visit to emergency clinic or health center or short hospitalization for a treatment of a medical condition was observed in 37.6% (n = 155) in the preceding 30 days before EWA causing a 2.4-fold increase in the risk of very high INR event compared to patients without EWA. Patients on chemotherapeutic agents had a 5.6-fold risk of overanticoagulation. EWA occurred during the first 6 months of warfarin treatment in 21.2% of patients in the EWA group. The number of patients with EWA per 6 months of warfarin treatment after the initiation of warfarin is presented in Fig 2.

Use of carbamazepine, amiodarone, dronedarone or tramadol was recorded in only 28 (6.8%) patients in the EWA group and 11 (2.7%) in the control group. Individually they had no independent predictive value in the multivariate analysis. SSRI/SNRI use was of borderline significance in predicting EWA. There was no statistical difference between groups in the use of paracetamol or simvastatin.

### Lifestyle and independence related factors

A total of 239 (58.0%) patients with EWA and 76 (18.8%) control patients had risk factors associated with lifestyle or were dependent on help from other people in everyday life. The lifestyle related significant predictors were active smoking, and excessive alcohol consumption. Alcohol abuse was observed in 17.7% of patients in the EWA Group and only 1.5% of control patients, predicting EWA with an odds ratio of over 20.

## Discussion

Non-vitamin K antagonist oral anticoagulants (NOACs) are increasingly used as the first choice for anticoagulation in patients with AF, but warfarin is still widely used and remains the only option for patients with mechanical valve prosthesis [12–14]. Our study shows that severe overanticoagulation is a rare phenomenon during warfarin treatment and can be predicted with many patient characteristics together with temporary predisposing factors highlighting the complexity and multifactorial etiology of excessive anticoagulation. By identifying these risk factors, it is possible to improve the safety of warfarin anticoagulation.

Our results show that even though long-term warfarin treatment exposes to overanticoagulation over time, the first months after introduction of the treatment carry the highest risk (Fig 1). This finding is in line with earlier reports on risks of major bleeding and overanticoagulation during warfarin treatment [2, 15–17]. AF is detected and anticoagulation often initiated in an acute setting such as exacerbation of chronic disease or acute decompensated heart failure. Therefore, early dosing may be affected by concomitant medications, or patients may not be accustomed to nutrients affecting vitamin K metabolism.

Excessive alcohol consumption was associated with the greatest (24-fold) individual risk of EWA, reflecting the many problems of alcohol abuse with warfarin treatment: direct alcohol inhibition of warfarin breakdown, impairment of liver function and problems in drug compliance [18]. Active smokers were also found to be at risk of overanticoagulation and as with alcohol abuse, addiction treatment may prove useful also in stabilizing INR levels. Given the obvious reasons for EWA in patients with alcohol abuse, a critical assessment of patient’s net clinical benefit of anticoagulation therapy should be undertaken. Theoretically, some of these patients could benefit from NOACs instead of warfarin use, but this concept has not been proven in clinical trials nor in real world practice.

It is well known that in patients with heart failure, malignancies and chronic kidney diseases an episode of very high INR may reflect the overall disease burden and exacerbations of the underlying disease. Spontaneous INR elevations are often present at the end stage of a critical illness and the high INR is a consequence of the disease process rather than a specific problem with warfarin. Reflecting this background, chronic heart failure was very common among EWA patients. Even though heart failure is an established risk factor for ischemic stroke in AF patients, congestion may impair liver function and reduce the synthesis of coagulation factors thus predisposing also to excessive anticoagulation [19]. In addition to heart failure, advanced age and active cancer have been reported to prolong the return of elevated INR to therapeutic level [20].

Chronic kidney disease is a common finding among AF patients and a major risk factor for a bleeding event, cardiovascular and all-cause mortality also in patients with only mild renal impairment [21–25]. Expectedly, severe renal dysfunction was also a strong predictor of EWA. This result highlights the need for careful assessment of indications and bleeding risks when considering anticoagulation for patients with AF and severe renal impairment. For patients with AF on hemodialysis, the bleeding risks are often thought to outweigh the benefits of oral anticoagulation [26, 27]. This reasoning is supported by our current findings, as all our study patients with kidney failure (eGFR <15) suffered an EWA event.

Malignant diseases are known to be prothrombotic, but in patients on warfarin, active cancer also seems to increase the risk of overanticoagulation. Nevertheless, only a minority of patients in this study had an active malignancy. This can be partly explained by the use of low molecular weight heparin in cancer patients, although there is no clear recommendation on its use among AF patients in contrast to patients with deep vein thrombosis [28]. In patients with mechanical valve prosthesis the risk of EWA was high (15-fold) and may be a result of a higher target INR level than in patients with AF. One possible explanation may be excessive dose elevation due to fear of thromboembolic complications in subtherapeutic INR levels, although the actual target INR is only slightly higher than that for AF patients. In fact, this finding is in line with the recent publication by Grzymala-Lubanski et al reporting a higher incidence of bleedings, death and total complications related to higher warfarin intensity in comparison to lower intensity of anticoagulation [14, 29].

In accordance with previous studies, antibiotic or antifungal treatments were strong predictors of EWA and all study patients with antifungal medication experienced EWA [30, 31]. More frequent INR controls together with critical prescription policy might help to avoid these events or at least to detect them earlier. Concomitant use of known interactive medication should be avoided to reduce the risk of INR instability and bleeding events [32, 33]. The low rates of interactive medication in our study may be explained by the national electronic database for interactive medication (Swedish, Finnish, Interaction X-referencing -database) used widely in clinical practice in Finland. Interestingly, cholesterol-lowering drugs were found to make an EWA event 50% less likely. This is in accordance with a previous smaller report on statin therapy protecting warfarin treated patients against overanticoagulation[17].

Outpatient emergency visits and hospitalizations were also independent risk factors for severe overanticoagulation. While the above mentioned chronic underlying diseases/conditions may affect the level of warfarin anticoagulation, it is also possible that physicians tend to adjust warfarin dosage based on incidental INR values at the hospital without appropriate knowledge on long-term dosing or drug adherence. Furthermore, these treatment episodes may also include procedures during which warfarin discontinuations may be necessary and the following reinitiation of warfarin potentially exposes to overanticoagulation. These findings highlight the need of frequent outpatient INR controls in these acute settings together with better patient education.

It was not a surprise that patients who are institutionalized or otherwise dependent on outsider help in everyday life were also at risk of EWA. This may be a reflection of worse overall health status or non-adherence to medication [34]. Even though the simple dosage of NOACs might prove more practical in this subgroup, the detection of overanticoagulation might be more difficult compared to warfarin treatment with regular INR controls.

The Turku University Hospital laboratory service provider (TYKSLAB) has exceptionally comprehensive follow-up data coverage with virtually all INR samples analyzed by the same laboratory regardless of the place of residence in the southwestern Finland. Also, people living in this region have no tendency for migration enabling reliable and comprehensive follow-up data. This unusual setting provides invaluable data for our analysis. Even though the electronic patient records have good coverage, we were reliant on the clinical data and diagnoses documented by the physicians and other medical professionals taking care of the patient. Information on the level of adherence to medication or warfarin dosage on patient records was scarce and thus not registered. To ensure the stability of INR in the control group patients, we used a method, which favors the selection of patients with longer treatment durations. For this reason, the comparison of treatment duration between groups was not presented. The selection of patients with the least INR elevations on long-term warfarin anticoagulation (Control group) will by definition favor patients with low-risk for complications.”

## Conclusions

Our large retrospective study shows that the rare and complex complication of severe warfarin anticoagulation can be predicted with several clinical factors. Identifying the risk factors may be helpful in prevention and early detection of episodes of very high INR.
